# Monitoring of Non-Ferrous Wear Debris in Hydraulic Oil by Detecting the Equivalent Resistance of Inductive Sensors

**DOI:** 10.3390/mi9030117

**Published:** 2018-03-08

**Authors:** Lin Zeng, Hongpeng Zhang, Qiang Wang, Xingming Zhang

**Affiliations:** 1 Marine Engineering College, Dalian Maritime University, Dalian 116026, China; bobzl@dlmu.edu.cn; 2 Shanghai Salvage Ministry of Transport, Shanghai 200000, China; wq@cose.cn; 3 School of Naval Architecture and Ocean Engineering, Harbin Institute of Technology, Weihai 264209, China; zhxm@hit.edu.cn

**Keywords:** non-ferrous wear debris, micro inductive sensor, hydraulic oil, equivalent resistance method

## Abstract

Wear debris in hydraulic oil contains important information on the operation of equipment, which is important for condition monitoring and fault diagnosis in mechanical equipment. A micro inductive sensor based on the inductive coulter principle is presented in this work. It consists of a straight micro-channel and a 3-D solenoid coil wound on the micro-channel. Instead of detecting the inductance change of the inductive sensor, the equivalent resistance change of the inductive sensor is detected for non-ferrous particle (copper particle) monitoring. The simulation results show that the resistance change rate caused by the presence of copper particles is greater than the inductance change rate. Copper particles with sizes ranging from 48 μm to 150 μm were used in the experiment, and the experimental results are in good agreement with the simulation results. By detecting the inductive change of the micro inductive sensor, the detection limit of the copper particles only reaches 70 μm. However, the detection limit can be improved to 48 μm by detecting the equivalent resistance of the inductive sensor. The equivalent resistance method was demonstrated to have a higher detection accuracy than conventional inductive detection methods for non-ferrous particle detection in hydraulic oil.

## 1. Introduction

Hydraulic machinery is widely used in civil and military industries. As the blood of the hydraulic system, hydraulic oil not only has the effect of transmitting energy, but can also reduce friction between components and reduce the system temperature. Wear debris in hydraulic oil contains important information on the operation of the equipment, which is important for condition monitoring and fault diagnosis in mechanical equipment [1]. The detection of hydraulic oil can avoid the possibility of catastrophic component failure during operation [2]. Wear debris is one of the main causes of hydraulic mechanical failure [3,4]. During normal machine operations, the concentration of wear debris in the hydraulic oil remains the same and the particle size is small, typically in the range of 10–20 µm. When abnormal wear occurs, the debris concentration gradually increases, and the size of the debris particles increases to as large as 50–100 µm [5,6]. Statistics show that more than 80 percent of catastrophic failures are caused by particle contamination in the hydraulic oil [7]. Among these particles, 75 percent are metallic, which cause almost all failures [8,9]. The wear debris in the oil is an important information carrier for machinery wear. Wear debris, especially non-ferrous wear debris, indicates the key component of being worn. Many components in hydraulic systems contain non-ferrous metallic material, such as the copper slippers in hydraulic axial piston pumps, white metal linings, tin base white metal linings and the leading white metal linings, which include varieties of non-ferrous metal material. The wear debris will be oxidized if the particles remain in the oil for a long time, which will cause serious damage to the equipment. However, in the early stages of wear, most of the particles still have metallic properties [10]. Therefore, the monitoring of non-ferrous wear debris is essential for initial prediction of hydraulic system failures.

A few oil condition monitoring devices have been developed in recent years [11,12,13]. Optical methods, such as light blockage counters, are capable of detecting small particles in hydraulic oil [14]. However, the accuracy of the light blockage method is affected by fluid clarity, the particle refractive index and the existence of air bubbles. The acoustic emission detection method, which is based on the amplitude change of reflected acoustic waves, is sensitive to the influence of background acoustic emissions and lubrication oil temperature variations [15]. Rosenkranz et al. developed an electrical resistivity method to test the solid–solid contact ratio in order to detect catastrophic failure in tribological contacts [16] and performed some experimental studies using the wear particle analysis of stainless steel surfaces with periodic cross-like patterns [17]. Capacitive Coulter counting is very simple, but the measured capacitance change often reflects not only the presence of particles but also the changes in lubricant properties, such as the viscosity and total acid number [18]. However, none of these methods can distinguish between ferrous and non-ferrous metallic particles. Du Li developed an inductive counter counting device for wear debris detection [19,20,21]. Furthermore, the improvement of the coil structure, the external amplification circuit, and the inductance-capacitance (LC) resonance method enhanced the sensitivity [22,23,24,25,26]. By detecting the inductance change, the inductive sensors, which are widely used in the field for oil particle detection, can distinguish between ferromagnetic and non-ferromagnetic metal particles. However, the inductance sensor has a lower sensitivity to non-ferromagnetic particles (such as copper particles). Previous studies have shown that the detection limit of inductive sensors for copper particles is 125 μm, and by adding a complex LC resonant circuit to the inductive sensor, the detection limit can theoretically reach 55 μm [27]. 

Compared with the traditional inductive sensor, the microfluidic chip-based inductive sensor has a higher detection accuracy. Instead of detecting the inductance change of the inductive sensor, in this paper, the equivalent resistance change of the inductive sensor was detected for non-ferrous particle (copper particles) monitoring based on our 3-D micro solenoid inductance sensor [28], which was published previously. Both the simulation and the experiment results show that the resistance change is more sensitive than the inductance change for non-ferromagnetic metal particle detection.

## 2. Sensor Design and Detection Principle

**Figure 1:**
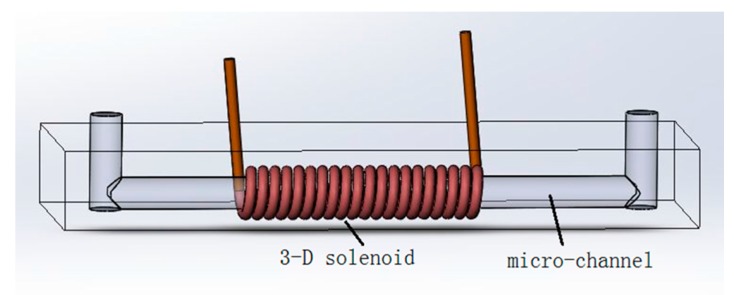
Design of the micro inductive sensor for hydraulic oil detection: the diameters of the solenoid coil and the micro-channel are 25 µm and 300 µm, respectively.

The micro inductive sensor is shown in Figure 1. It consists of a 3-D solenoid coil, which is the core of the microfluidic inductive sensor, and a micro-channel made using the mold construction method. The main purpose for the mold construction method in building a micro-channel instead of a glass tube is to decrease the distance between the solenoid coil and the particle. In this way, the micro inductive sensor is more sensitive [29]. To build the 3-D solenoid, we first prepared a small steel wire (300 µm in diameter) with its surface polished smooth. Then, a 600 turn 3-D solenoid coil was built by carefully winding the fine copper line (25 µm in diameter, with a thin insulation) around the small steel wire. After the solenoid was formed, a small amount of polydimethylsiloxane (PDMS) was applied to the 3-D solenoid coil and dried in a thermostat to fix the fine copper line. Then, the small steel wire was removed using pincers to form the micro-channel. Finally, the oil sample inlet was made using a punch. The reason for building a 600 turn 3-D solenoid coil instead of a two-layer planar coil was to strengthen the magnetic field and enhance the density of the magnetic flux at the center of the coil.

An alternating current is applied across the 3-D solenoid coil, which induces an alternating magnetic field in the sensor. The impedance of the sensor is calculated by

(1) Z=R+jωL

where *j*^2^ = −1, *Z* is the impedance of the coil, *R* and *L* are the resistance and inductance of the coil, respectively, and *ω* is the angular frequency of the alternating current. The impedance *Z* is determined by the alternating magnetic field. When the oil containing metallic wear debris passes through the sensor, the magnetic field is changed due to the influence of the metallic particles. As a result, the impedance *Z*, as well as the resistance *R* and the inductance *L*, are also changed.

Due to high permeability, the ferrous metallic particles are magnetized in a high-frequency magnetic field, and the magnetizing field of the particle is in the same direction as the original magnetic field, so the total magnetic flux is enhanced [30]. Compared with the magnetic field of the eddy currents, the magnetizing field of the magnetization is much larger, and the effect of any eddy currents can be ignored, so the coil equivalent inductance will be increased. As a result, a positive inductive change and a positive resistance change will be induced by a ferrous metal particle.

**Figure 2:**
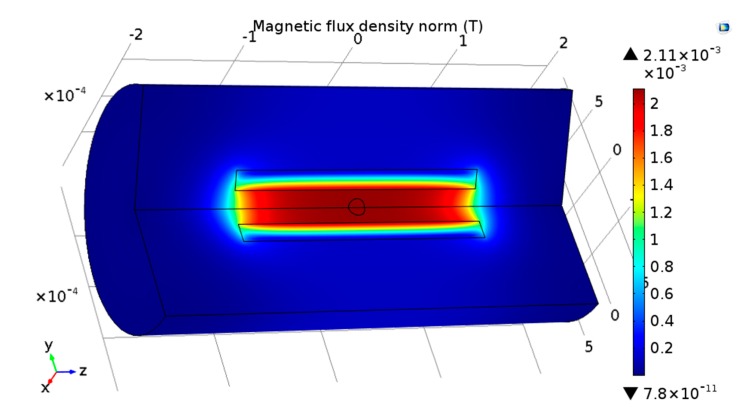
Magnetic field distribution within the sensor as influenced by the non-ferrous metallic particle.

When a non-ferrous metallic particle passes through the center of the coil, there is no magnetization, but an eddy current will be generated inside the particle due to the alternating magnetic field. The magnetic fields from the eddy currents will offset some original magnetic fields, further affecting the magnitude and phase of the current in the solenoid. As a result, the total magnetic flux of the coil will decrease [31], leading to a decrease in the coil inductance and an increase in the coil equivalent resistance. As the AC excitation frequency and eddy currents increase, large decreases in the equivalent inductance and increases in the equivalent resistance are observed. Thus, a negative inductive change and a positive resistance change are induced by a non-ferrous metallic particle. The magnetic field change induced by the non-ferrous metallic particle was simulated using COMSOL software (COMSOL Multiphysics 5.0, COMSOL Inc., Stockholm, Sweden), as shown in Figure 2. The model structure parameters are consistent with the design parameters of the sensor. Non-ferrous metallic particles (copper particles, for example) with different sizes were used to simulate the resistance and inductance changes in the coil. These results are shown in the next section for comparison with the experimental results.

## 3. Experiments and Discussions

### 3.1. Experimental Procedure

**Figure 3:**
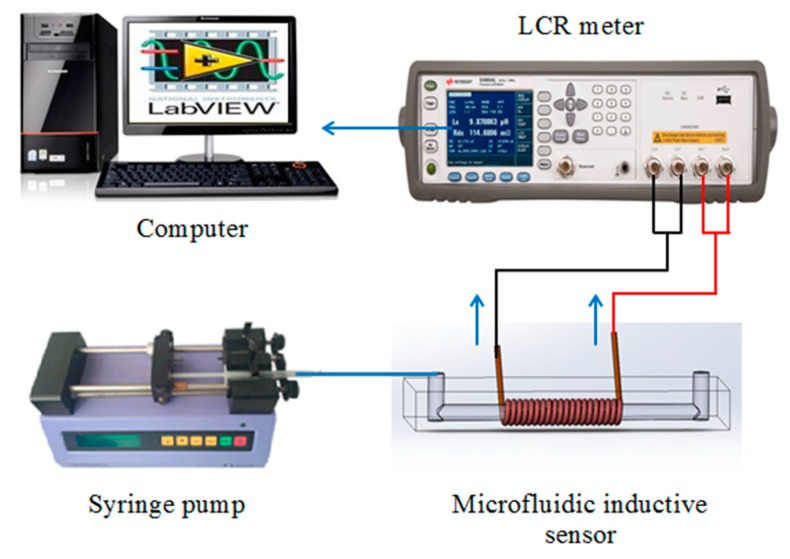
The impedance detection system.

The experimental system is illustrated in Figure 3. It is composed of a syringe pump (Harvard Apparatus B-85259, Harvard Apparatus, Holliston, MA, USA), a microfluidic inductive sensor, an LCR impedance analyzer (Agilent E4980A, Agilent Technologies Inc., Bayan Lepas, Malaysia) and a computer. Copper particles with different sizes are used to test the detection system. A plastic pipe is used to connect the injection pump and the detection chip. The oil sample is injected into the micro-channel and then flows through the center of the solenoid at a controllable velocity. The volume flow of the oil sample is set at 0.04 mL/min. The LCR meter is connected to the solenoid with an AC excitation applied to it. Therefore, the resistance and inductance of the 3-D solenoid coil can be monitored by the LCR meter. In all experiments, the excitation signal applied to the LCR meter was a 2 MHz, 2 V sine wave. The LCR meter was set up to assume that the solenoid coil consists of a pure resistance and inductance in series, and the resistance and inductance of the coil can be detected by the LCR meter simultaneously. When there are no metal particles in the hydraulic oil, the basic resistance and inductance are approximately 61.2 Ω and 54.9 μH, respectively.

Roughly spherical copper particles (Hefei Shatai Mechanical and electrical technology Co., Ltd., Hefei, China) with different sizes were used in the experiments. A series of steel sieves was used to select copper particles with sizes ranging from 48 µm to 53 µm, 58 µm to 62 µm, 65 µm to 74 µm, 80 µm to 86 µm, 90 µm to 96 µm, 96 µm to 106 µm and 150 µm to 160 µm, within these size ranges, the size of the particles is evenly distributed. In the experiments, the particles with different size were mixed with the corresponding hydraulic oil (The Great Wall L-HM 46, Sinopec Lubricant Co., Ltd., Beijing, China) to create different oil samples with 100 mL of oil and 4 mg of copper particles. These oil samples were injected into the micro-channel using a syringe pump to start the experiments. 

### 3.2. Results and Discussions

**Figure 4:**
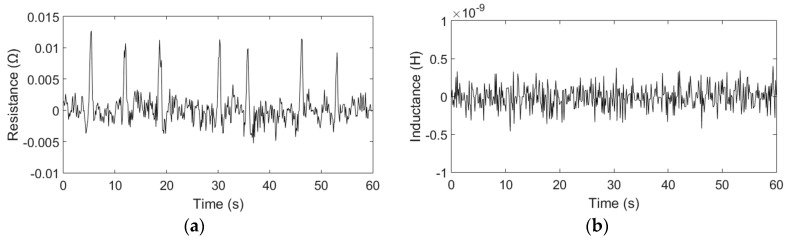
Detection results of the copper particles with sizes ranging from 48 µm to 53 µm: (**a**) Resistance detection results; (**b**) Inductance detection results.

**Figure 5:**
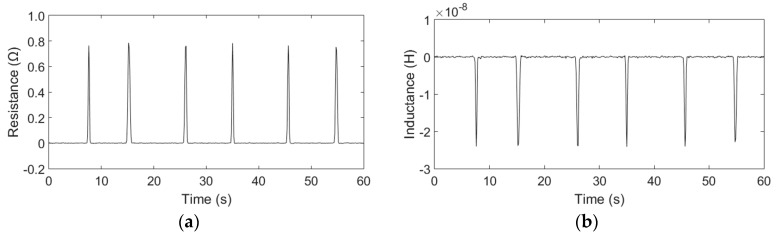
Detection results of the copper particles with sizes ranging from 150 µm to 160 µm: (**a**) Resistance detection results; (**b**) Inductance detection results.

The detection results are shown in Figure 4 and Figure 5.

Figure 4 shows the resistance and inductance changes generated by the small copper particles (48–53 µm). In the experiments, the sensor has detected 29 particles (48–53 µm) in 5 min using the equivalent resistance method (due to space constraints, we did not put all the detection results in the manuscript), but two of the signals were barely identifiable in the signal diagram, so based on the detection results, the probability of detecting 48–53 µm particles is 93.1%. When we detected the 42–48 µm copper particles, there were only 3 identifiable signals in 5 min, so we considered that the detection limits of the sensor is 48 µm copper particles. The average amplitude of the resistance is approximately 0.01 Ω; however, the inductance change cannot be detected because the particle size is too small, and the signal is lost within the noise. The resistance and inductance detection results of the copper particles with sizes ranging from 150 µm to 160 µm are shown in Figure 5. The detection results show that when copper particles flow through the sensor, positive resistance changes and negative inductive changes were observed. Each signal change represents the passage of one copper particle. The average amplitudes of the resistive changes and the inductance changes are approximately 0.76 Ω and 2.36 × 10^−8^ H, respectively, and their signal-to-noise ratios (SNRs) are approximately 152.7 and 47.2, respectively. The comparison results show that the equivalent resistance change is more sensitive to particle size than the inductance change for non-ferrous metallic particle detection. For the small copper particles, the equivalent resistance method has a lower detection limit than inductance method. Thus, the smaller particles can be detected using the resistance method. For the relatively large copper particles, the equivalent resistance method also has a higher SNR than the inductance method. 

**Figure 6:**
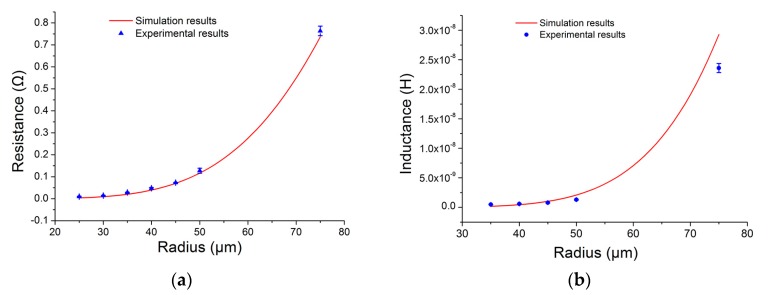
Comparison of the experimental results and the simulation results: (**a**) Detection results of the copper particles with diameters ranging from 48 µm to 150 µm; (**b**) Detection results of the copper particles with diameters ranging from 70 µm to 150 µm.

Next, by calculating the average of 10 detected signal amplitudes for particles of each size, and comparing them with the simulation results, the experimental results were verified. The comparison results are shown in Figure 6.

Figure 6 shows that the experimental results are in good agreement with the simulation results. Both the resistance and inductance changes increase with increasing diameter of the copper particles. In addition, the copper particles with sizes as small as 48 µm can be detected using the equivalent resistance method. However, the inductance method can only detect the copper particles with sizes large than 70 µm. Therefore, the sensitivity and accuracy can be improved by detecting the equivalent resistance of the coil. This is of great significance to optimize micro inductive sensors and to enhance the detection precision and accuracy.

## 4. Conclusions

In this paper, we demonstrated that an equivalent resistance method improves the sensitivity of a micro inductive sensor for non-ferrous wear debris detection. Instead of monitoring the inductance change in the micro inductive sensor, the equivalent resistance change of the sensor was monitored to detect non-ferrous wear debris in hydraulic oil. The resistance and inductance changes induced by the non-ferrous metallic particles were simulated first using COMSOL software. Then, resistance and inductance detection experiments were performed using copper particles with sizes ranging from 48 µm to 150 µm. The experimental results verify the simulation results. Compared with the 70 µm detection limit of the inductance method, we successfully detected 48 µm copper particles using the equivalent resistance method. Additionally, both the sensitivity and the SNR of the micro inductive sensor were significantly improved. The new method supports the improvement of micro inductance sensor accuracy, which is of great significance to the rapid detection of non-ferrous wear debris in hydraulic oil.
